# A metastatic distal-type epithelioid sarcoma: Case report and review

**DOI:** 10.1016/j.ijscr.2020.04.096

**Published:** 2020-05-16

**Authors:** Avalon Regalbuto, Andrew Tudosie, Eveline Klenotic

**Affiliations:** a Ohio University Heritage College of Osteopathic Medicine, Cleveland, 4180 Warrensville Center Road, Warrensville Heights, OH 44122, United States; b Lake Health West Medical Center, 36000 Euclid Ave, Willoughby, OH 44094, United States

**Keywords:** Epithelioid, Sarcoma, SMARCB1, Metastasis, Biomarker

## Abstract

**Introduction:**

Epithelioid sarcoma is known as one of the rarest types of sarcomas and was identified as its own diagnosis by Dr. Franz Enzinger in 1970 after his realization of its massive overlap with many other diseases. This tumor has an aggressive clinical course with high recurrence and metastasis rates.

**Presentation of case:**

This report will detail the case of a 39-year-old male who was diagnosed with Epithelioid Sarcoma and later succumbed to this disease.

**Discussion:**

This report will emphasize epithelioid sarcoma morphology and immunohistochemistry with discussions on predisposition, prognostic factors, and current options for treatment modalities.

**Conclusion:**

Future studies are needed to determine clear predisposition and screening practices; however modern pharmaceuticals have shown hopes of optimizing the course of this cancer.

## Highlights

• Epithelioid Sarcoma is a rare soft tissue tumor with an aggressive nature.
• Certain biomarker positivity or negativity can help distinguish from other tumors.
• SMARCB1 loss is predominant in most Epithelioid Sarcomas.
• Wide surgical excision is main treatment, but biomarker-targeted therapy is growing.

## 1 Introduction

Epithelioid sarcoma is an extremely rare soft tissue tumor, occurring in less than 1% of soft tissue sarcomas [1,2]. They most commonly occur in younger males [[1], [2], [3]]. And present as a painless, nodular mass in either the dermis, subcutaneous tissue, or along tendinous or fascial planes [[1], [2], [3]]. There are two types: distal-type epithelioid sarcoma and proximal-type epithelioid sarcoma, based solely on anatomic location. It is not enough to differentiate these based on location alone, but instead determining their histopathological features too [1,3]. Microscopically, they both express epithelioid morphology [1,3]. However, the proximal-type will express cytologic pleomorphism, nuclear atypia, and even signet-ring-like vacuolation, with characteristic rhabdoid morphology and absent pseudo-granulomatous pattern [1,4]. Whereas the distal-type will commonly have spindled or sarcomatous appearance in the periphery of the mass, with centrally scattered necrosis [1,3].

Epithelioid sarcomas were most commonly confused with synovial sarcomas until Dr. Enzinger realized these tumors didn’t fit that exact picture [5]. And even with biopsy, it is still commonly misdiagnosed by its resemblance to granulomatous disease, chronic inflammation, and epithelial neoplasia [1,2,5]. Therefore, its workup relies heavily on histopathology, demographics, location, and immunohistochemistry to narrow its differential [1,2].

The intended purpose of this case report is to discuss the genetic predisposition, prognosis, and treatment, and ultimately aid as an educational tool for optimal patient-centered care. This work has been reported in line with the SCARE criteria [6].

## 2 Presentation of case

In 2016, a 39-year-old male presented to the general surgery outpatient office with a three-month history of a right-hand mass at the base of thumb and a right axillary mass. The hand lesion had caused him pain and numbness of the hand and lateral aspect of the second digit. The axillary lesion had caused him a pulsatile, sharp pain when he laid down. Previously, the hand lesion had been attributed to scar tissue, until the axillary mass appeared, to which more workup was pursued. MRI of the brachial plexus was performed and showed a 5.3 × 9.4 × 8.8 cm lobulated right axillary mass and involvement of the right lateral chest wall inferior to the right brachial plexus. It demonstrated increased heterogenous, focal nodular intensity, and scattered internal non-enhancement, suggestive of necrosis. An MRI of the right hand showed a heterogenous soft tissue mass, near the median nerve. An excisional biopsy confirmed metastatic epithelioid sarcoma from a distal-type tumor at the right hand. Immunohistochemistry showed positivity for tumor biomarkers AE1, AE3, EMA, vimentin, and CD34 and negativity for INI1 and S100. When treatment was discussed, he declined all therapy. CT showed metastasis to lung and suspicious metastasis to thoracic, lumbar, and sacral spine. The patient succumbed to his disease three months later.

## 3 Discussion

Epithelioid sarcomas are a mixture of both mesenchymal cells and epithelial cells [1,2,7]. And thus, when immunohistochemistry is performed, there will be positive tests for both tissue types. In other words, CD34 (50%), vimentin, and EMA (85%) are most commonly positive in epithelioid immunohistochemistry, which aids in ruling out malignant rhabdoid tumors [1,2,7,8].

It is also common to see negative biomarkers: S-100, CD31, and SMARCB1/INI1. Up to 90% of epithelioid sarcomas have a homozygous loss of SMARCB1/INI1 expression [1,2,7,8], confirming a two-hit carcinogenesis [4]. However, SMARCB1 cannot be used alone in finalizing a diagnosis because they are also negative in malignant rhabdoid tumors [4]. It has also been discussed that ERG, SALL4, and glypican-3 (GPC3) can also be analyzed to differentiate between malignant rhabdoid tumors (MRT) and epithelioid sarcomas (ES) [4]. “In one study, ERG was negative in all cases of MRT and positive in 25% of proximal-type ES and SALL4 was positive in two-thirds of MRT but only rarely in ES. In another study, GLPC3 was overexpressed in over 40% of MRT but negative in nearly all ES [4].” *Refer to Table* Table 1*1* [4,7].

**Table 1:** Biomarker expression in Epithelioid Sarcoma compared to Malignant Rhabdoid Tumor.

	CD34	VIMENTIN	EMA	INI1	S100	ERG	SALL4	GPC3	CD31
Epithelioid Sarcoma	+	+	+	–	–	+	–	–	–
Malignant rhabdoid tumor	–	+	+	–	+	–	+	+	–

Overall, there is incredible overlap between malignant rhabdoid tumors and epithelioid sarcomas [8], putting into question the heritable nature of epithelioid sarcomas, creating uncertainty in whether these two tumors should remain categorically distinct [4,9].

Malignant rhabdoid tumors are considered hereditary SWI/SNF complex deficiency syndromes [10], which describes a chromatin-remodeling complex dysfunction that affects gene expression, proliferation, and differentiation [10]. One of its major subunits is SMARCB1, which is usually completely lost [8,10] by either deletion in epithelioid sarcomas or point mutations in malignant rhabdoid tumors [7]. Accordingly, once the complex is lost, its tumor suppressor capabilities are gone, rendering cancer growth uncontrolled.

Although these tumors are similar, there may be a strong possibility that the age at which SMARCB1 inactivation occurs is what dictates the earlier onset and predisposition to malignant rhabdoid tumors versus epithelioid sarcomas [9].

Thus, in patients with epithelioid sarcoma, genetic screening is not indicated, as family history has not been shown to be a risk factor. Instead, epithelioid sarcomas etiology remains unknown, posing a potential cause as scar tissue or trauma [2,3,5].

The ability to determine prognosis at the time of diagnosis can dictate the quality of life and intervention. According to a clinicopathological analysis of 106 epithelioid sarcomas: having a proximal-type tumor, a tumor size greater than 5 cm, tumor multifocality, and high mitotic index had a shorter five-year survival rate; additionally, tumors greater than 5 cm and a high mitotic index had a shorter five-year metastasis-free survival rate [8]. Similar findings were seen in a 1985 retrospective review of 241 epithelioid sarcomas with additional prognostic factors such as deeper depth, nodal involvement, necrosis, and vascular invasion [3]. Interestingly, neural invasion was less likely to metastasize than vascular invasion [3], which is linked to sarcomas hematogenous spread [11].

It is also worth noting that in the past, it was advised against using a grading system for epithelioid sarcomas. But recently, FNCLCC grading system established prognostic value [9]. There is a statistically significant association between higher grades and SMARCB1-negative tumors, but no statistically significant association between SMARCB1-status and epithelioid sarcoma subtype, stage, or location [9]. Similarly, it is noted that SMARCB1-negative epithelioid sarcomas have more recurrence and metastasis than SMARCB1-positive tumors (76% versus 47%) [9]. Lastly, older age and male gender can worsen prognosis [2,3].

Given the aggressive nature of this tumor, early surgical management is the treatment of choice [[1], [2], [3]], where radical resection has 10–12% local recurrence rates and non-radical resection has 36–60% recurrence rates [[12], [13], [14]]. Interestingly, even in the most invasive treatments like amputation, survival and recurrence rates didn’t prove superior to successful radical resection [12].

Chemotherapy has recently shown progress, especially adriamycin, vincristine, cytoxan, actinomycin D, and methotrexate [3]. While systemic administration has provided satisfactory palliation, its response is of short duration [2,15] whereas localized, neoadjuvant chemotherapy limits systemic toxicity and shows signs of decreasing tumor size [12]. Additionally, verapamil and cyclosporine A could increase sensitivity to chemotherapy to reverse multidrug-resistance [16,17]. And sunitinib, a tyrosine kinase inhibitor, has shown reasonable disease stabilization as well [16,18]. Similarly, radiation therapy has shown mixed effects as it can lower risk of local recurrence with preoperative and/or postoperative radiation therapy combined with radical surgery [12] or not [12,19,20].

Recently, a wave of biomarker-targeted therapy has provided new opportunities to tackle treatment, especially targeting AKT/mTOR pathway. Losing SMARCB1 leads to hyperactivation of the AKT/mTOR pathway, which is involved in cell proliferation and cancer survival [16]. Everolimus, an mTOR inhibitor, has shown a delay in tumor growth, but not any reduction in tumor size [16]. Instead, using a combination of agents to block AKT and c-MET has shown to be more effective in inducing tumor arrest [16]. Similarly, EGFR helps upregulate cyclin D1 and HGFR/MET pathway to sustain AKT activation, enhancing tumor growth [16]. New combinations such as erlotinib and rapamycin, which suppress both EGFR and mTOR, could cause tumor cell cycle arrest and apoptosis [16].

Lastly, in a current clinical trial, an oral EZH2 inhibitor, tazemetostat, has shown a 26% disease control rate, defined as any duration of stable disease lasting ≥ 32 weeks [21]. EZH2 is an epigenetic modulator and oncogenic driver when SMARCB1 is lost [21]. And so, with tazemetostat on the rise, healthcare can potentially add this to the epithelioid sarcoma treatment regime.

## 4 Conclusion

Epithelioid sarcoma is a rare soft tissue tumor that poses a challenge due to its aggressive nature and broad morphology. Certain tumor biomarkers have even been identified as either present or absent on immunohistochemistry. It is important to determine predisposition, prognosis, and treatment once diagnosed, with wide surgical excision as the mainstay and new therapeutic techniques on the horizon.

## Declaration of Competing Interest

The authors declare that there are no conflicts of interest regarding the publication of this article.

## Sources of funding

The authors declare that there was no involvement of funding for this research.

## Ethical approval

This study has been exempt from ethical approval by Lake Health West Medical Center.

## Consent

Written and signed consent has been obtained; statement of consent is present at the end of manuscript.

## Author contribution

• **Avalon Regalbuto** : study concept and design, obtainment of patient consent, data collection, literature search, writing and editing of the paper, manuscript preparation for publication
• **Andrew Tudosie** : obtainment of patient consent, data collection, literature search, writing and editing of the paper, manuscript preparation for publication
• **Eveline Klenotic** : obtainment of patient consent, editing of the paper

Author roles have been further defined by the CRediT criteria on Page 1 of the manuscript.

## Registration of research studies

1. Name of the registry: Research Registry
2. Unique identifying number or registration ID: researchregistry5395
3. Hyperlink to your specific registration (must be publicly accessible and will be checked): https://www.researchregistry.com/browse-the-registry#home/registrationdetails/5e5c299f55b3d20019743b8c/

## Guarantor

Guarantor for this manuscript: **Avalon Regalbuto**.

## Provenance and peer review

Not commissioned, externally peer-reviewed.

## CRediT authorship contribution statement

**Avalon Regalbuto:** Conceptualization, Investigation, Resources, Writing - original draft, Writing - review & editing, Visualization, Supervision, Project administration. **Andrew Tudosie:** Investigation, Resources, Writing - original draft, Writing - review & editing, Visualization. **Eveline Klenotic:** Investigation, Writing - review & editing.
